# Supplementary figures and images for: Development of primary osteoarthritis during aging in genetically diverse UM-HET3 mice
Source: Arthritis Res Ther. 2024 Jun 8;26:118. doi: 10.1186/s13075-024-03349-y (PMC11161968; doi:10.1186/s13075-024-03349-y)

Supplement Figure 1

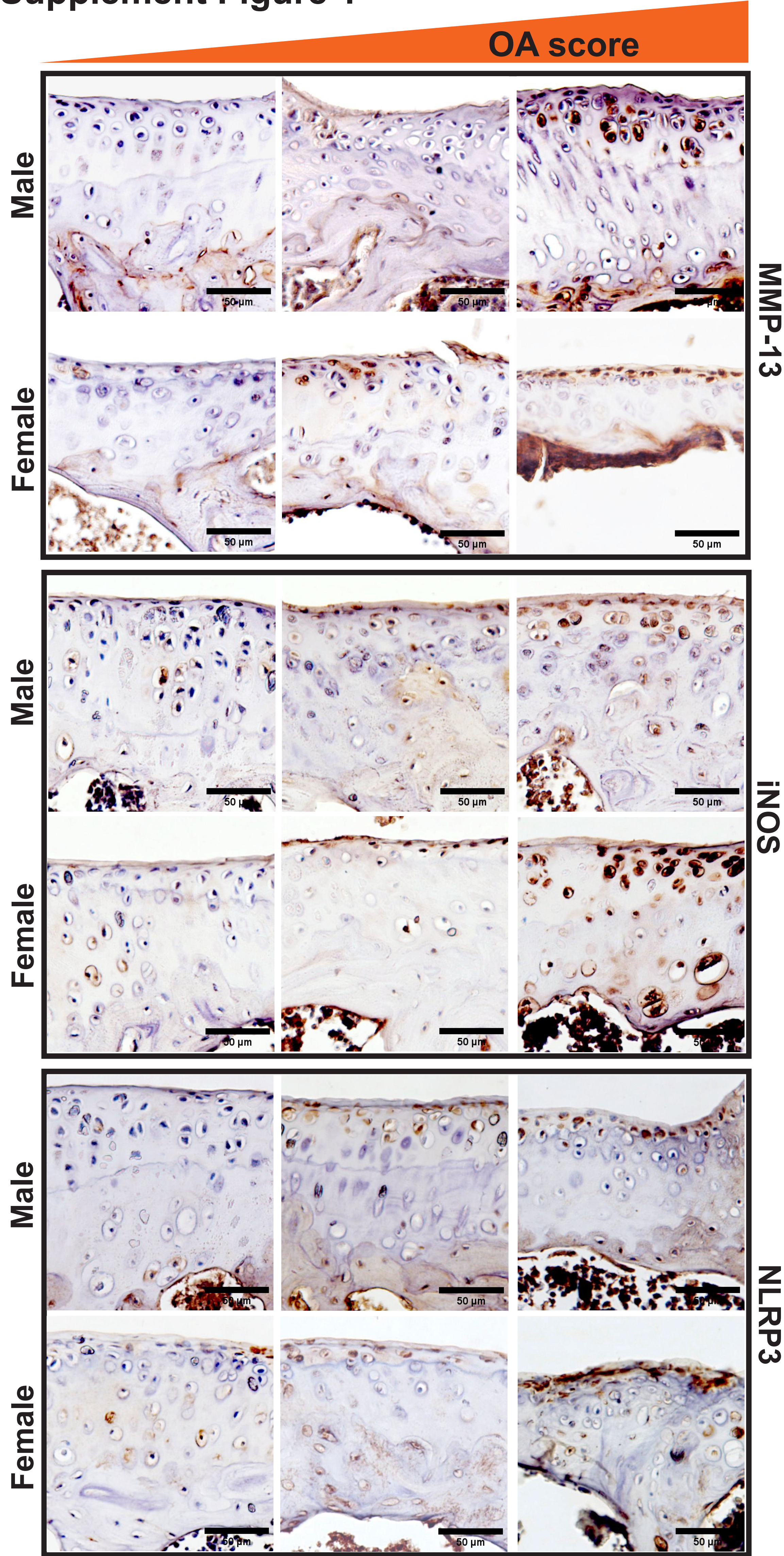

Supplement: Supplementary file 1 — Additional file 1: Supplement Figure 1. Shown are representative knee joint sections stained with MMP-13, iNOS, and NLRP3 antibodies in both male and female mice. Quantification is available in figure 4. [file 13075_2024_3349_MOESM1_ESM.pdf]

Supplement Figure 2

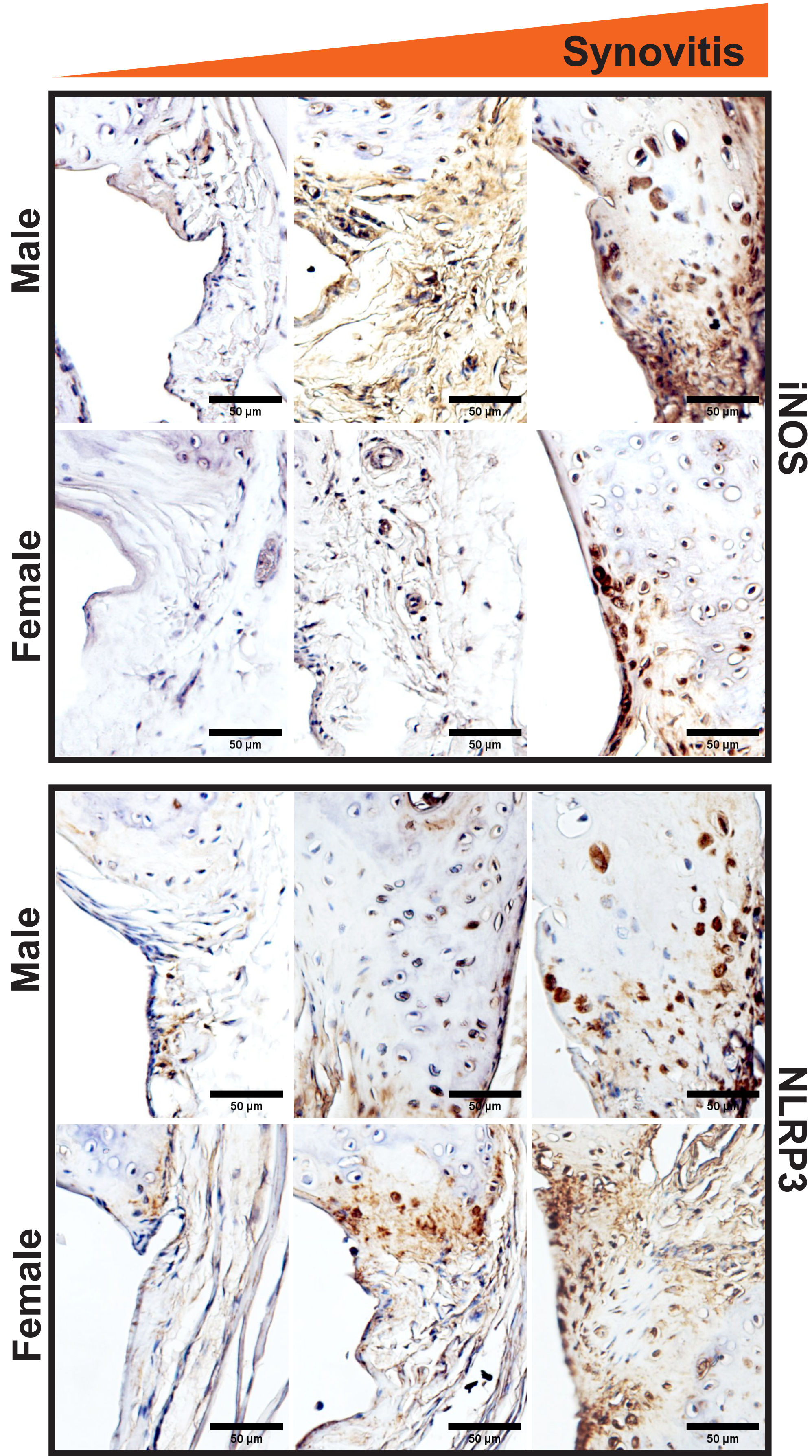

Supplement: Supplementary file 2 — Additional file 2: Supplement Figure 2. Shown are representative medial synovial membrane sections stained with iNOS and NLRP3 in both male and female mice. Quantification is available in figure 4. [file 13075_2024_3349_MOESM2_ESM.pdf]

Supplement Figure 3

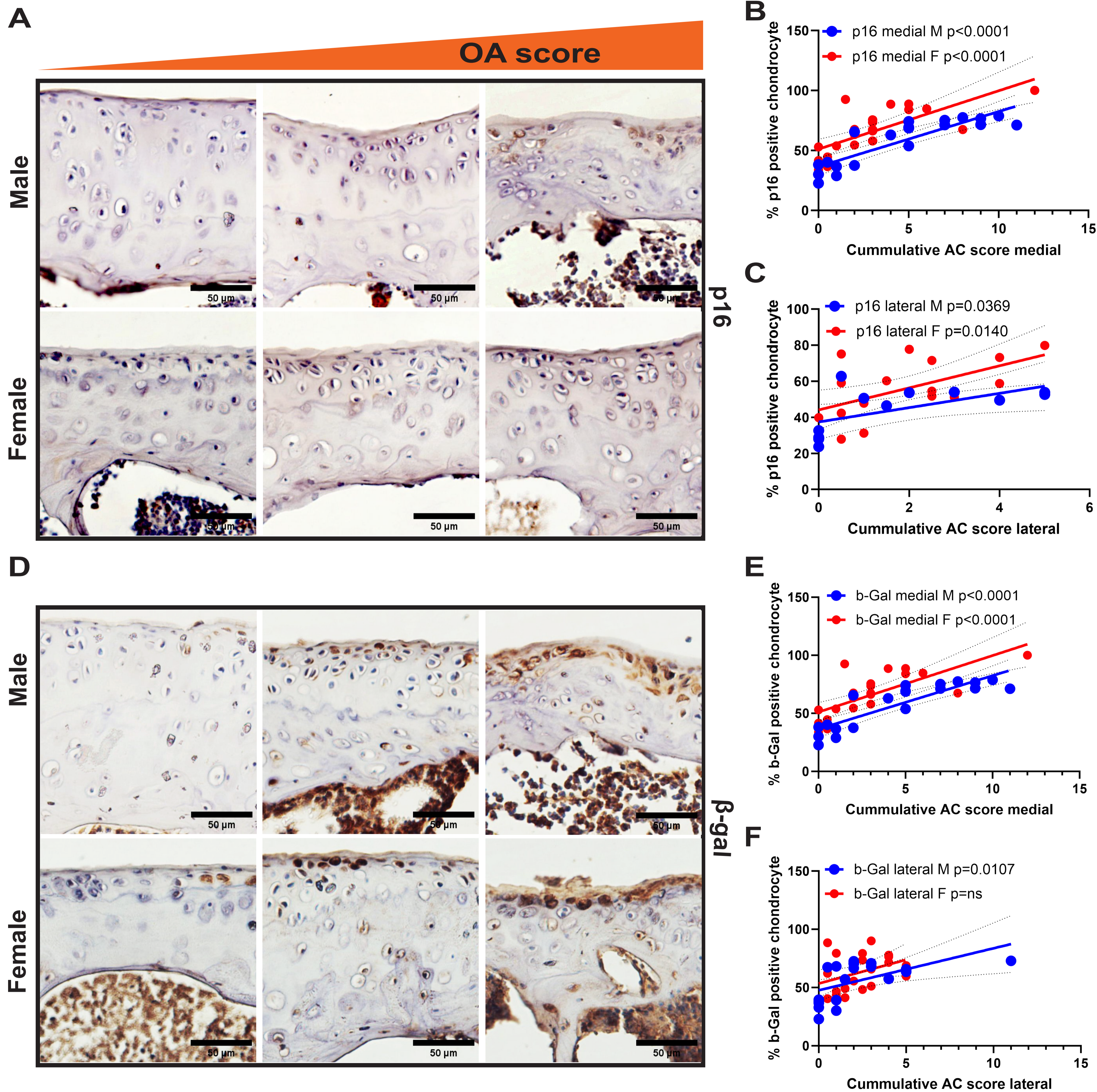

Supplement: Supplementary file 3 — Additional file 3: Supplement Figure 3. Shown are representative knee joint sections stained with p16 (A) and quantification of positive chondrocytes in the (B) medial and (C) lateral side of the tibia. (D) Representative knee joint sections stained with b-Gal and quantification of positive chondrocytes in the (E) medial and (F) lateral side of the tibia in both male and female mice. Males n=19 and females n=19. [file 13075_2024_3349_MOESM3_ESM.pdf]

Correlations between femur and tibia SCB traits

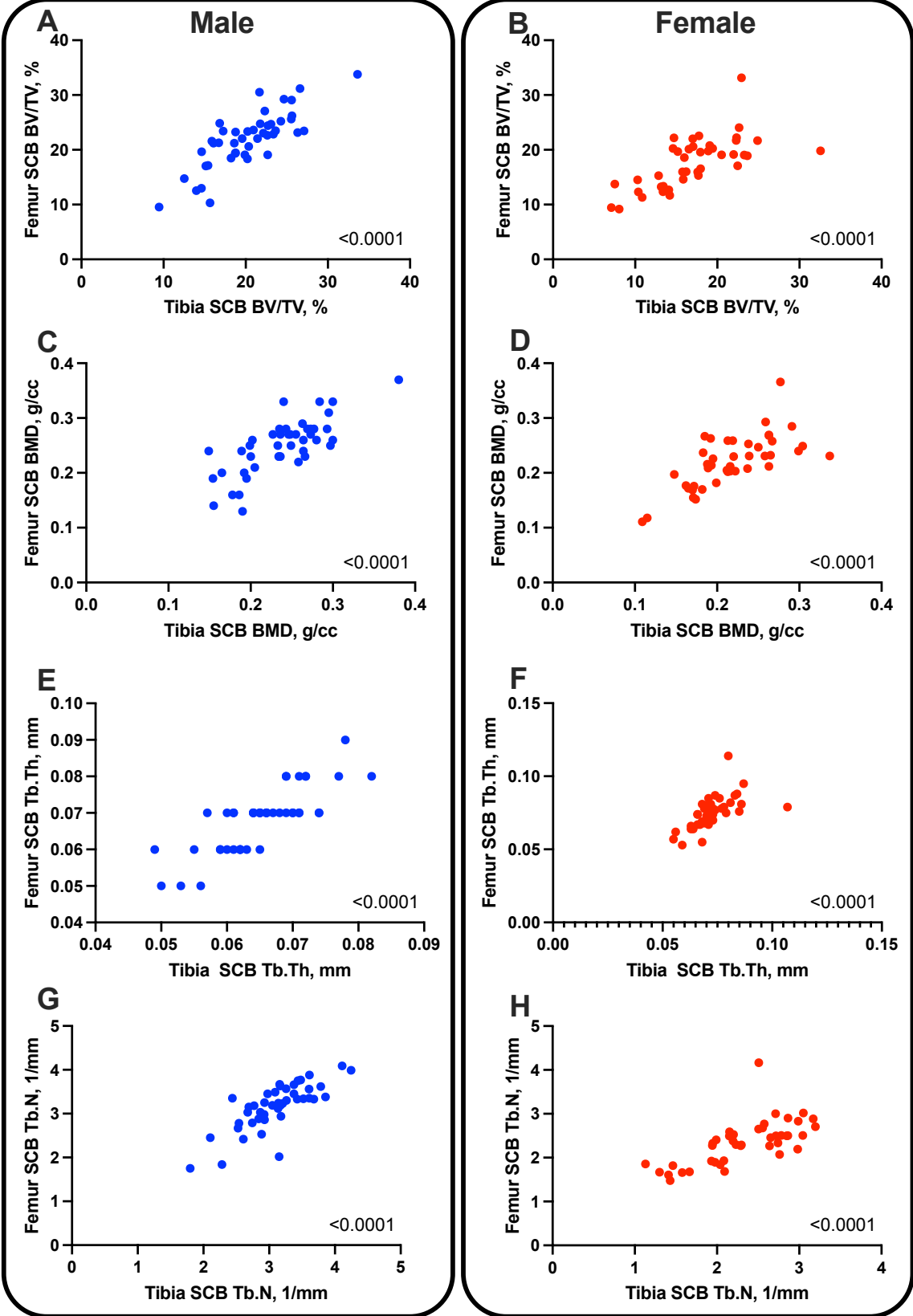

Supplement: Supplementary file 5 — Additional file 5: Supplement Figure 5. Femur and tibia SCB traits obtained by micro-CT are significantly directly correlated. Spearman’s rank correlations between SCB BV/TV (A,B), SCB BMD (C,D), SCB Th. (E,F), and SCB Tb.N (G,H) of femur and tibia in male and female mice. Malesn=47, Females n=45. P values are indicated for each correlation. [file 13075_2024_3349_MOESM5_ESM.pdf]

AC score at the medial and lateral sides of the tibia  
in relation to SCBP morphology

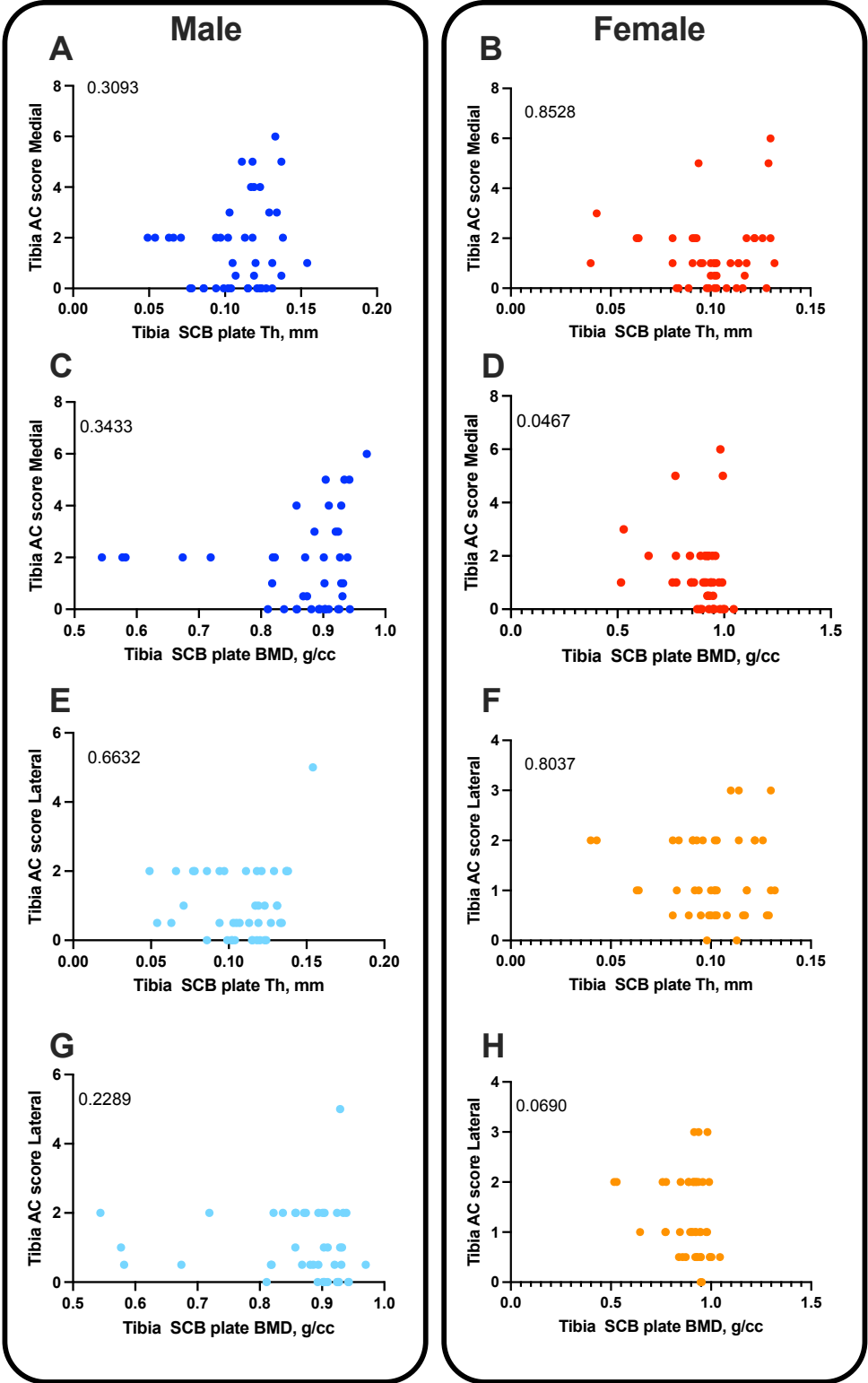

Supplement: Supplementary file 6 — Additional file 6: Supplement Figure 6. Cumulative AC (cAC) scores of the medial or lateral tibia show no correlation to the morphology of the SCBP by micro-CT. Spearman’s rank correlations between cAC scores at the medial tibia with SCBP Th (A,B) or SCBP BMD (C,D) in male and female mice. Similar correlations were done between cAC scores at the lateral tibia with SCBP Th (E,F) or SCBP BMD (G,H) in male and female mice. Males n=47, Females n=45. P values are indicated for each correlation. [file 13075_2024_3349_MOESM6_ESM.pdf]
