# Supplementary material for: Development of primary osteoarthritis during aging in genetically diverse UM-HET3 mice
Source: Arthritis Res Ther. 2024 Jun 8;26:118. doi: 10.1186/s13075-024-03349-y (PMC11161968; doi:10.1186/s13075-024-03349-y)

Supplement Figure 4

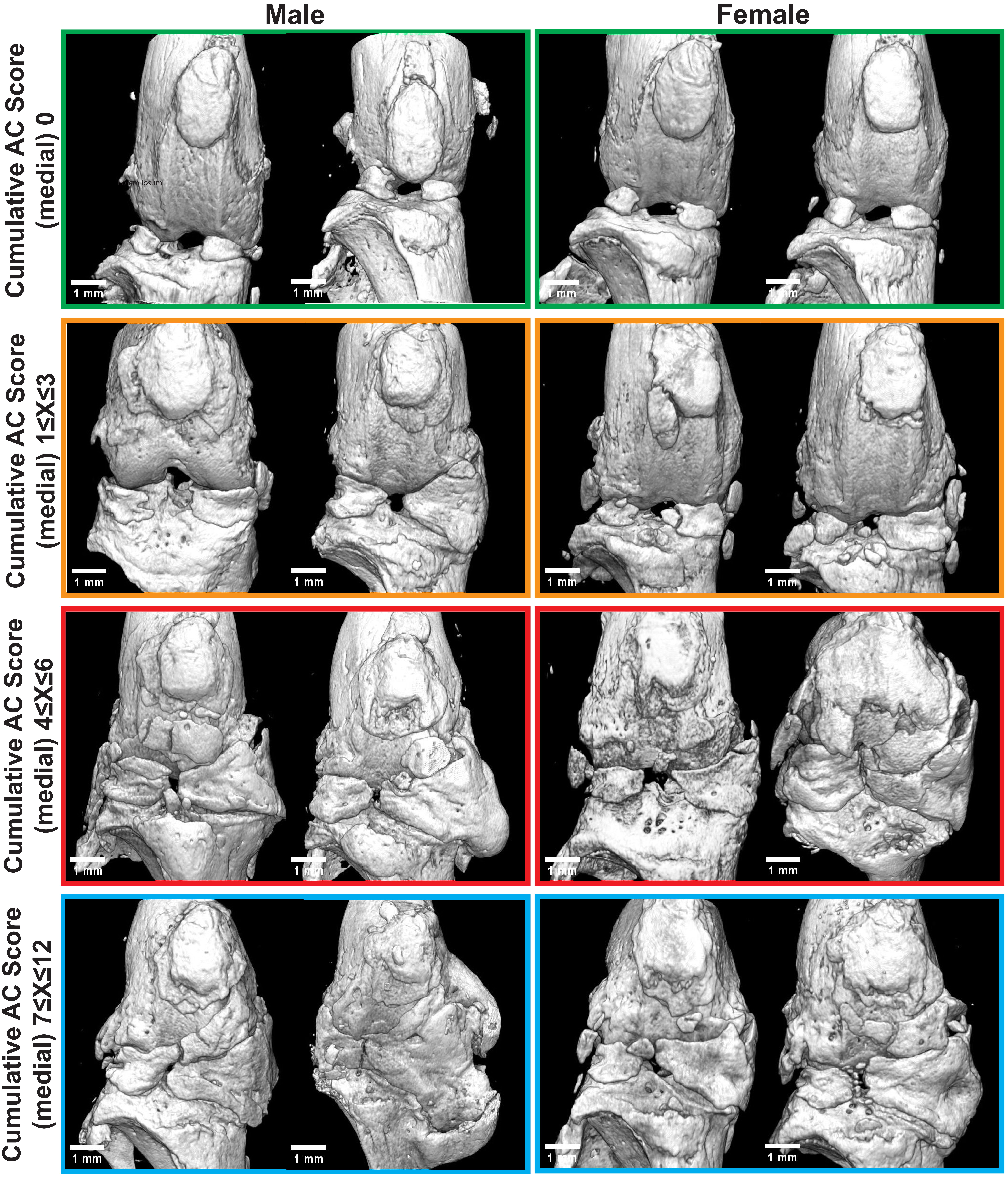

Supplement: Supplementary file 4 — Additional file 4: Supplement Figure 4. (A) 3D images of a micro-CT scan of knee joints from male and female mice with different OA severities (cumulative scores at the medial side of the joint). A link to a short movie presenting 3D reconstruction of knee joints with no histological evidence of OA or with high cumulative AC score obtained by histology (Mendeley Data, V1, doi: 10.17632/6nddwstfw3.1). [file 13075_2024_3349_MOESM4_ESM.pdf]
