# Supplementary material for: Development of primary osteoarthritis during aging in genetically diverse UM-HET3 mice
Source: Arthritis Res Ther. 2024 Jun 8;26:118. doi: 10.1186/s13075-024-03349-y (PMC11161968; doi:10.1186/s13075-024-03349-y)
Supplement: Supplementary file 7 — Additional file 7: Supplement Figure 7. A summary of the AC, osteophyte maturity, synovitis, and ectopic chondrogenesis scores at the whole joint, the medial or lateral side of the joint. (A) Cumulative AC (cAC) scores of the whole joint (medial+lateral tibia+femur) in control (CTL) MB-, or MQ-treated male and female mice. (B) cAC scores of the medial joint (medial tibia+femur) in control (CTL) MB-, or MQ-treated male and female mice. (C) cAC scores of the lateral joint (lateral tibia+femur) in control (CTL) MB-, or MQ-treated male and female mice. (D) Cumulative osteophyte maturity scores of the whole joint (medial+lateral tibia+femur) in control (CTL) MB-, or MQ-treated male and female mice. (E) Cumulative osteophyte maturity scores of the medial joint (medial tibia+femur) in control (CTL) MB-, or MQ-treated male and female mice. (F) Cumulative osteophyte maturity scores of the lateral joint (lateral tibia+femur) in control (CTL) MB-, or MQ-treated male and female mice. (G) Cumulative synovitis scores of the whole joint (medial+lateral) in control (CTL) MB-, or MQ-treated male and female mice. (H) Synovitis scores at the medial side of the joint in control (CTL) MB-, or MQ-treated male and female mice. (I) Synovitis scores at the lateral side of the joint in control (CTL) MB-, or MQ-treated male and female mice. (J) Cumulative ectopic chondrogenesis (Ect.Chon) scores of the whole joint (medial+lateral) in control (CTL) MB-, or MQ-treated male and female mice. (K) Ect.Chon scores at the medial side of the joint in control (CTL) MB-, or MQ-treated male and female mice. (L) Ect.Chon scores at the lateral side of the joint in control (CTL) MB-, or MQ-treated male and female mice. [file 13075_2024_3349_MOESM7_ESM.pdf]

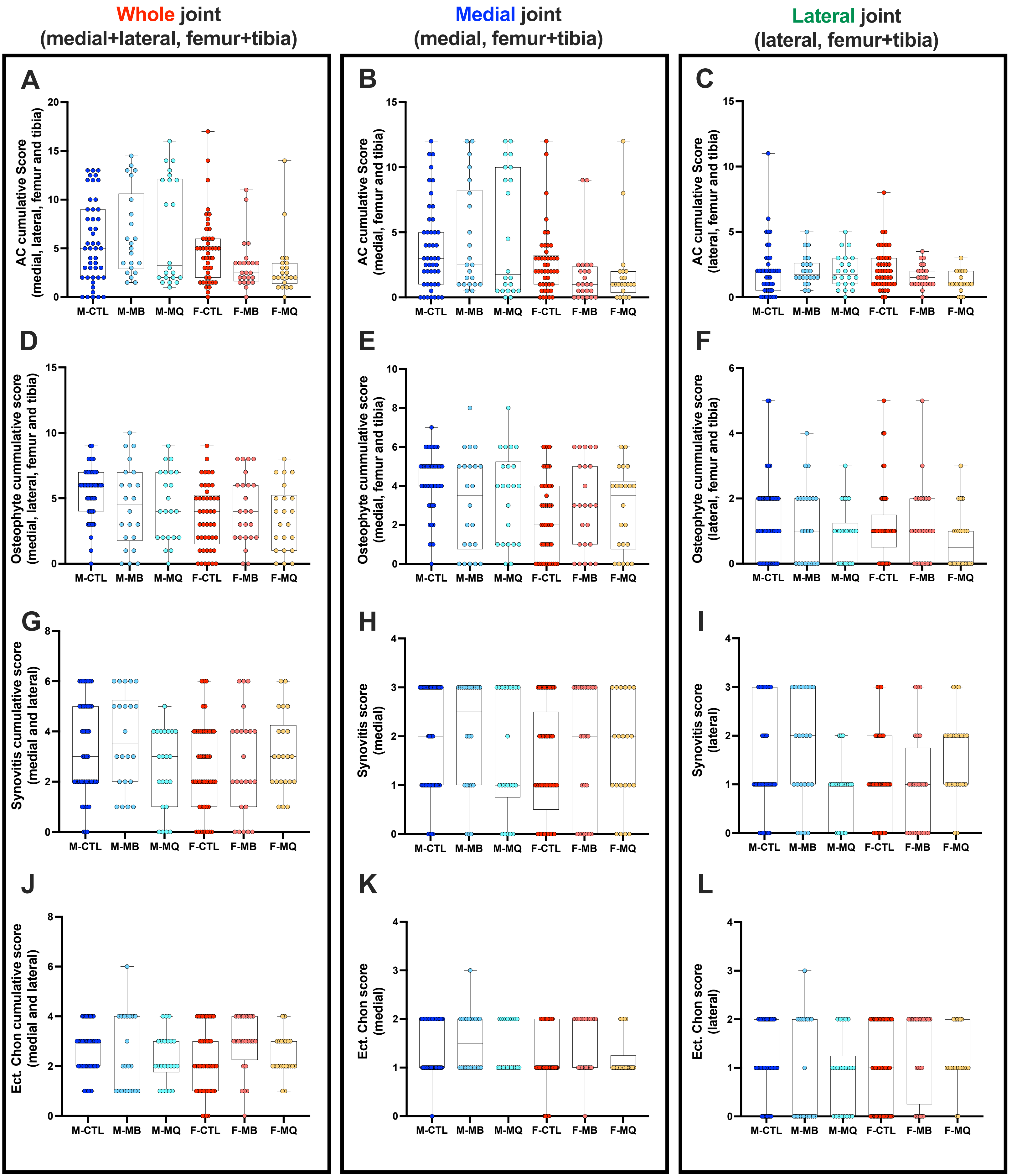

Whole joint (medial+lateral, femur+tibia)
Medial joint (medial, femur+tibia)
Lateral joint (lateral, femur+tibia)
A
B
C
AC cumulative Score (medial, lateral, femur and tibia)
AC cumulative Score (medial, femur and tibia)
AC cumulative Score (lateral, femur and tibia)
D
E
F
Osteophyte cummulative score (medial, lateral, femur and tibia)
Osteophyte cummulative score (medial, femur and tibia)
Osteophyte cummulative score (lateral, femur and tibia)
G
H
I
Synovitis cumulative score (medial and lateral)
Synovitis score (medial)
Synovitis score (lateral)
J
K
L
Ect. Chon cumulative score (medial and lateral)
Ect. Chon score (medial)
Ect. Chon score (lateral)
M-CTL
M-MB
M-MQ
F-CTL
F-MB
F-MQ
