# Supplementary material for: Development of primary osteoarthritis during aging in genetically diverse UM-HET3 mice
Source: Arthritis Res Ther. 2024 Jun 8;26:118. doi: 10.1186/s13075-024-03349-y (PMC11161968; doi:10.1186/s13075-024-03349-y)
Supplement: Supplementary file 8 — Additional file 8: Supplement Figure 8: Representative H&E-stained images of the synovial membrane at the mid-coronal region of the knee joint in both male and female mice. Magnification is at 200X, with scale bars of 150µm and 50µm. A red arrow indicates vascularization, a blue arrow represents fibrosis, and 'OC' denotes ossification in the synovium. [file 13075_2024_3349_MOESM8_ESM.pdf]

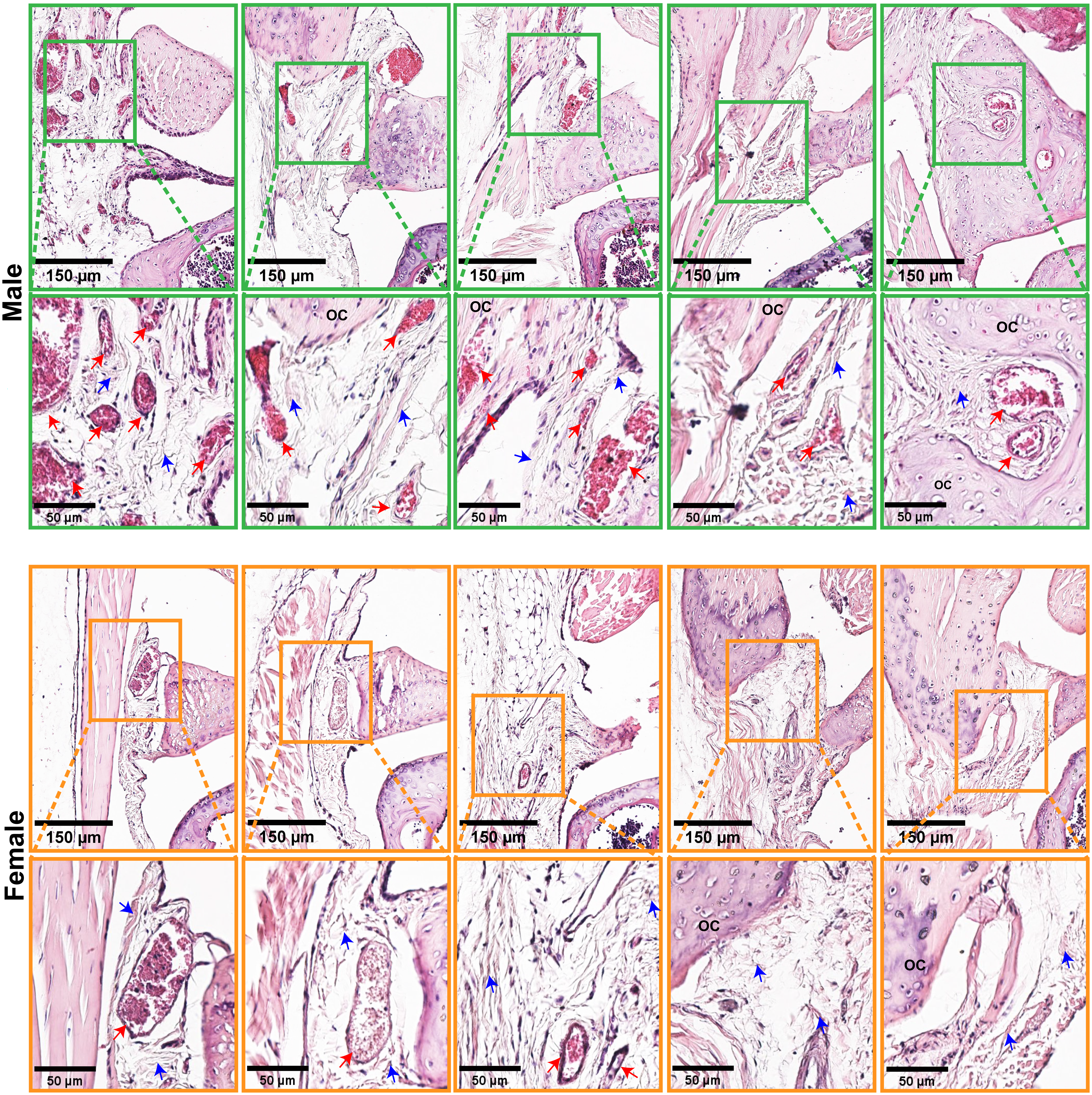
Male
Female
150 μm
50 μm
OC
oc
